# Synergizing metabolic flux analysis and nucleotide sugar metabolism to understand the control of glycosylation of recombinant protein in CHO cells

**DOI:** 10.1186/1472-6750-11-95

**Published:** 2011-10-18

**Authors:** Susan C Burleigh, Teun van de Laar, Corné JM Stroop, Wout MJ van Grunsven, Niaobh O'Donoghue, Pauline M Rudd, Gavin P Davey

**Affiliations:** 1 School of Biochemistry and Immunology, Trinity College Dublin, Dublin 2, Ireland; 2 Merck, Sharp & Dohme, Oss, Netherlands; 3 Glycobiology Lab, National Institute for Bioprocessing Research and Training, Fosters Avenue, Mount Merrion, Blackrock, Co. Dublin, Ireland

## Abstract

**Background:**

The glycosylation of recombinant proteins can be altered by a range of parameters including cellular metabolism, metabolic flux and the efficiency of the glycosylation process. We present an experimental set-up that allows determination of these key processes associated with the control of *N*-linked glycosylation of recombinant proteins.

**Results:**

Chinese hamster ovary cells (CHO) were cultivated in shake flasks at 0 mM glutamine and displayed a reduced growth rate, glucose metabolism and a slower decrease in pH, when compared to other glutamine-supplemented cultures. The *N*-linked glycosylation of recombinant human chorionic gonadotrophin (HCG) was also altered under these conditions; the sialylation, fucosylation and antennarity decreased, while the proportion of neutral structures increased. A continuous culture set-up was subsequently used to understand the control of HCG glycosylation in the presence of varied glutamine concentrations; when glycolytic flux was reduced in the absence of glutamine, the glycosylation changes that were observed in shake flask culture were similarly detected. The intracellular content of UDP-GlcNAc was also reduced, which correlated with a decrease in sialylation and antennarity of the *N*-linked glycans attached to HCG.

**Conclusions:**

The use of metabolic flux analysis illustrated a case of steady state multiplicity, where use of the same operating conditions at each steady state resulted in altered flux through glycolysis and the TCA cycle. This study clearly demonstrated that the control of glycoprotein microheterogeneity may be examined by use of a continuous culture system, metabolic flux analysis and assay of intracellular nucleotides. This system advances our knowledge of the relationship between metabolic flux and the glycosylation of biotherapeutics in CHO cells and will be of benefit to the bioprocessing industry.

## Background

The conformation, solubility, antigenicity, activity and recognition properties of any given glycoprotein may be influenced by the structure of its *N*-linked glycans [1]. Control of glycoslyation requires an understanding of the metabolic and cellular alterations that lead to modifications in these structures, in addition to the use of stable cultivation methods. For example, a reduction in the adenylate energy charge (a measure of the metabolic and physiological status of a cell) of fed-batch CHO cells was found to coincide with decreased site occupancy of recombinantly produced IFN-γ [2] In another case, although no link was observed between metabolic fluxes and glycosylation site occupancy in CHO cells cultivated in a continuous set-up with limiting glutamine or glucose, a reduction in the concentrations of nucleotides and amino sugars was found to limit the site occupancy of IFN-γ [3].

Use of continuous cultivation, metabolic flux analysis and assay of intracellular sugar nucleotide levels is an ideal method to study the control of *N*-linked glycosylation due to the stability of the culture mode. After a defined period of time, the viable cell number, productivity and concentrations and rates of consumption and production of metabolites stabilize in this set-up, and a steady state is attained. This is achieved with the continuous flow of fresh medium into a culture inoculated in a bioreactor, and this is coupled with outflow of harvest culture at the same rate. Additionally, the pH, temperature, pO_2_, pCO_2 _are controlled by the bioreactor set-up. The set-up for these experiments is based on previous literature where the effect of a range of pCO_2 _values on the glycosylation of IgG was firstly screened in a batch culture, and then scaled up to a more controlled culture environment [4]. A different large-scale culture method was used in the work described here (a continuous culture system where the cells freely collect in the harvest reservoir) but the same sequential experimental culture system was used. The aim of returning the culture to the initial conditions was to illustrate that the method of culturing had no impact on the results, and, to provide replicate data to prove this point.

This study aims to investigate the mechanisms of changing glycan microheterogeneity by coupling metabolic flux analysis to assay of intracellular sugar nucleotides. A CHO cell line recombinantly expressing HCG was initially cultivated in batch shake flasks since it presents an ideal small-scale method to test the effect of many parameters on the subsequent glycosylation of recombinant proteins. In this case, 0 mM, 4 mM or 8 mM glutamine was supplied in the culture medium and the structures of the *N*-linked glycans attached to HCG were determined on days 1, 3 and 5 of culture. The same cell line was subsequently inoculated in a continuous culture system, and the glutamine concentration in the culture medium was altered between 8 mM and 0 mM to study four steady states in total. Metabolic flux analysis was carried out at each steady state, and the intracellular content of nucleotides and sugar nucleotides and structure of *N*-linked glycans attached to HCG were also determined. With use of both of these cultivation systems, decreased glycolytic flux and intracellular concentration of UDP-GlcNAc was found to correlate with a reduction in the sialylation and antennarity of the *N*-linked glycans attached to HCG under glutamine-limited conditions.

## Methods

### Cell line and culture medium

A CHOK1 (Chinese hamster ovary) cell line recombinantly expressing HCG (human chorionic gonadotrophin) was a kind gift of Merck, Sharp & Dohme, Oss, Netherlands. The cells were routinely maintained in CD-CHO medium (GIBCO) supplemented with 8 mM glutamine and HT supplement (100 μM sodium hypoxanthine, 16 μM thymidine, GIBCO). Cells were precultured in shake flasks in a Kuhner ClimoShaker ISF1-X incubator at 150 rpm, 5% CO_2 _and 80% RH.

### Batch cultivation

Cells were inoculated at 0.5 × 10^6 ^cells/ml in a volume of 150 ml in 500 ml shake flasks. The cultures were inoculated to result in a final concentration of 0 mM, 4 mM and 8 mM glutamine (gln). Six shake flasks per condition were incubated, and duplicate cultures were harvested on days 1, 3 and 5 of culture.

### Continuous cultivation

Duplicate continuous cultures were carried out in 2L bioreactors (Applikon) with a working volume of 1.7L. Culture pH and dissolved oxygen were controlled at 7.20 and 50% of air saturation, respectively, with the use of an ADI 1030 bioprocessor (Applikon). The temperature was maintained at 37°C and agitation rate was set at 325 rpm. The bioreactors were inoculated at an initial cell density of 0.3 × 10^6 ^cells/ml and cultured for three days in batch mode. At this point, the harvest pump (Watson Marlow 101 U/R) was switched on in order to set the medium flow rate at 0.4/d. The culture level was maintained at 1.7 L with use of a level controller (AppliSens APS501 level controller), which activated the delivery of fresh medium to the bioreactor via the feed pump when necessary. Fresh, sterile medium was stored at 4°C, while harvest cell suspension was collected into a reservoir stored at room temperature. (At each steady state the cell suspension was harvested at 4°C via a clean harvest line). A theoretical steady state is attained 5 volume changes after the last perturbation, and if there were minimal changes in the mean value of each cell culture parameter at this point, a steady state was defined. The glutamine concentration was altered between values of 8 mM and 0 mM to study four steady states in total, defined as follows; days 30-33 (state 1, 8 mM glutamine), days 45-48 (state 2, 0 mM glutamine), days 72-75 (state 3, 8 mM glutamine) and days 88-91 (state 4, 0 mM glutamine).

### Routine culture analysis

Viable cell number and cell viability was determined using the CEDEX AS^20 ^(Roche Innovatis AG), the extracellular concentrations of ammonium, glucose, glutamate, glutamine and lactate were assayed with use of the Nova Bioprofile 100 plus (Nova Biomedical Corporation) and BGA parameters (pH, pO2, and pCO2) were determined with the Bayer Rapidlab 248 (Siemens Healthcare Diagnostics). Cell-free supernatants were stored at -20°C before analysis of HCG content by ELISA at key time points.

### Calculation of metabolic parameters

The specific rate of substrate consumption (qS) and product formation (qP) and was calculated as described below (equations 1 and 2, respectively), i.e. the rate of change in substrate or product concentration (-d[S] and d[P], respectively) per unit time (dt) per viable cell number (more correctly, the average viable cell number between these time points, VC_ave_):

(1) $$qS=\frac{-d\left[S\right]}{dt}\cdot \frac{1}{VC_{ave}}$$

and

(2) $$qP=\frac{d\left[P\right]}{dt}\cdot \frac{1}{VC_{ave}}$$

Consequently, rates of substrate consumption and product formation were plotted as positive values. The chemical degradation of glutamine was taken into account for calculation of the rate of glutamine consumption (equation 3) and ammonium production (equation 4) [5].

(3) $$qGln=\frac{-d\left[Gln\right]}{dt}\cdot \frac{1}{VC_{ave}}-k\cdot \left[Gln\right]$$

and

(4) $$qN{H_{4}}^{+}=\frac{d\left[N{H_{4}}^{+}\right]}{dt}\cdot \frac{1}{VC_{ave}}-k\cdot \left[Gln\right]$$

The value for the first order degradation rate constant, *k*, was determined for CD-CHO medium in both a shake flask and bioreactor system by incubating glutamine-supplemented medium under experimental conditions. The glutamine concentration was determined daily and a plot of the percentage glutamine remaining as a function of time was constructed, and fitted to the exponential function y = e^-*k.x*^. A value of *k *= 0.078/d and 0.069/d was determined for bioreactors and shake flasks, respectively.

### Metabolic flux analysis

On three consecutive days during each steady state, cell culture supernatant was harvested and stored at -20°C from each bioreactor. The concentration of extracellular amino acids was subsequently determined by HPLC (SAFC Biosciences). Specific rates of production of amino acids, Nova metabolites, product titer and biomass were combined with material balances of carbon and nitrogen via a reaction network to estimate the metabolic fluxes at each steady state. The excel algorithm version of a metabolic flux analysis model developed by Mr. Jongchan Lee in the University of Minnesota was used [6]. The oxygen uptake rate was determined by a dynamic method [7], and since the respiratory quotient was assumed to be 1, the oxygen uptake rate equaled the carbon evolution rate. The carbon balance was varied between each steady state, with a minimal average value of 85 ± 5% determined at state 3 and maximal average value of 96 ± 10% determined at state 2. Negative flux values (J) indicate that the net flux is in the opposite direction to that quoted (i.e. a negative value of Jphe-tyr indicates net production of phenylalanine from tyrosine).

### Assay of intracellular nucleotides and sugar nucleotides

Cells were harvested for analysis on two consecutive days during each steady state by adaptation of the technique of Kochanowski *et al *[8]. Firstly, the length of incubation of the cell pellets in perchloric acid was increased to 10 minutes. The elution gradient on a Supelcosil LC-18-DB column was as follows: 0% B for 17 minutes (0.5 ml/min), 0-30% B for 27 minutes (1 ml/min), 30% B for 5 minutes (1 ml/min) and 0% B for 20 minutes (0.5 ml/min), where B represents the buffer described in the cited publication. Co-elution of GDP-Man and UDP-Gluc was observed in CHO extracts under these conditions.

### Purification and analysis of N-linked glycans of HCG

HCG was purified from culture supernatant harvested on two consecutive days during a steady state by affinity chromatography. The structure of the *N*-linked glycans attached to 5 μg of purified HCG were determined according to the method of Royle *et al *[9]. Briefly, the glycans were released from SDS-PAGE gel pieces by PNGase F digestion and labeling with 2-aminobenzamide (2-AB) before analysis on NP-HPLC. The structures were digested with a number of exoglycosidase enzymes in order to assign structures with use of GlycoBase [10]. The following exoglycosidase enzymes were used; sialidase A (ABS, hydrolyses α2-6, 3, & 8 non reducing terminal sialic acids), β-galactosidase (BTG, cleaves β(1-3, 4) galactose), α-fucosidase (BKF, releases α(1-2, 3, 4, 6) fucose) hexosaminidase (GUH, cleaves non-bisecting βGlcNAc residues) and mannosidase (JBM, releases α(1-2, 3, 6) mannose residues).

### Statistical analysis

One-way ANOVA with Bonferroni's post-test was performed using GraphPad Prism version 4.0a for Macintosh (GraphPad Software, San Diego, California, USA). Statistical significance was illustrated as follows; * = p < 0.05, ** = p < 0.01, *** = p < 0.001.

## Results

### Cell growth

**Figure 1:**
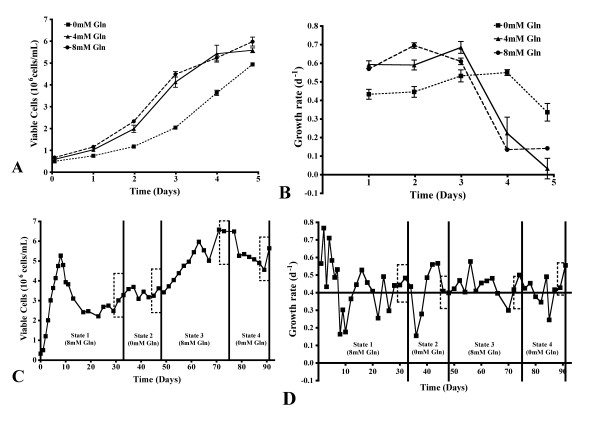
**Effects of glutamine on growth rates in batch shake flasks and bioreactor continuous culture**. Viable CHO cell number and growth rate with 0 mM, 4 mM and 8 mM glutamine supplementation in shake flask culture (A and B, respectively), and at varying glutamine concentrations in a bioreactor continuous culture system (C and D, respectively). Boxes are used to illustrate the steady state examined. Panels A and B, n = 6; panels C and D, n = 8.

On days 1 through 3 of shake flask cultivation at 0 mM glutamine, the number of viable cells was significantly lower than the glutamine supplemented cultures (p < 0.01, Figure 1A). However, the growth rate of the cells cultivated at 0 mM glutamine was only significantly lower than the glutamine supplemented cultures on day 2 (p < 0.01, Figure 1B). In addition, on days 4 and 5 of culture without glutamine, the growth rate remained higher than the glutamine supplemented cultures (p < 0.05).

Within the continuous cultivation, the number of viable cells increased significantly at steady states 3 and 4 (p < 0.001, 8 mM and 0 mM glutamine, Figure 1C). A flow rate of 0.4/d was chosen for this culture system since this represented a midway value between the observed minimal and maximal cell growth rate in batch culture. Although the harvest flow rate remained very close to this set point during the cultivation time (data not shown), a larger variation in the cell growth rate was observed (Figure 1D). However, statistical analysis indicates no difference in the cell growth rate at the defined steady state periods. There was no major glutamine-dependent change in the growth rate in this culture system since the growth rate was controlled by the rate of dilution of culture medium.

### Metabolism of glucose and lactate

**Figure 2:**
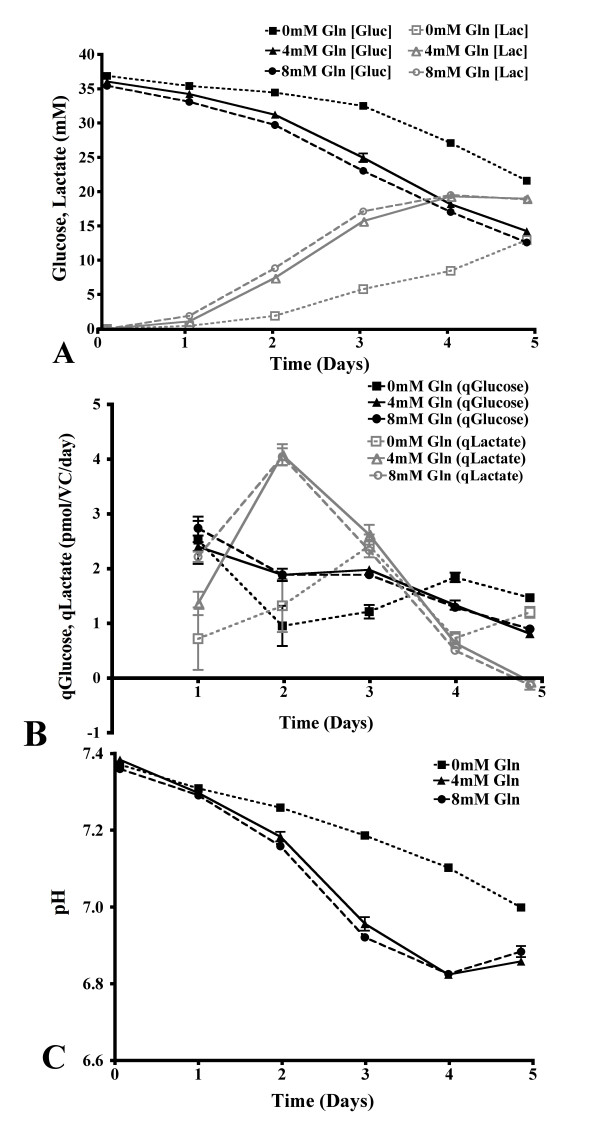
**The effects of glutamine on glucose, lactate and pH in batch shake flasks**. The extracellular concentration of glucose with 0 mM, 4 mM and 8 mM glutamine supplementation and lactate (identical symbols, colored grey) in CHO cell batch shake flask cultivation (A). The rates of glucose consumption and lactate production with batch shake flask cultivation at varying glutamine concentrations (symbols identical to previous, B). The extracellular culture pH during shake flask cultivation with 0 mM, 4 mM and 8 mM glutamine supplementation (C). Panels A, B and C, n = 6.

The initial concentration of glucose in both these set-ups was approximately 35 mM, the concentration provided in basal CD-CHO media. There was a significantly higher extracellular concentration of glucose in the glutamine-free shake flask culture, when compared to the glutamine supplemented cultures at all time points (p < 0.01, Figure 2A). This resulted in a lower concentration of lactate on days 2 through 5 of cell culture at 0 mM glutamine (p < 0.01, Figure 2A). On day 2 of culture without glutamine, the rate of glucose consumption and lactate production was significantly lower than the glutamine supplemented cultures (p < 0.05, Figure 2B). However, these rates were increased in the glutamine-free cultures on day 5 of cultivation (p < 0.01). The significantly reduced lactate concentration at 0 mM glutamine in the shake flask cultivation led to a slower decrease in the culture pH than the glutamine supplemented cultures, with a decrease from 7.4 to 7.0 noted from day 0 to day 5 of culture (Figure 2C).

**Figure 3:**
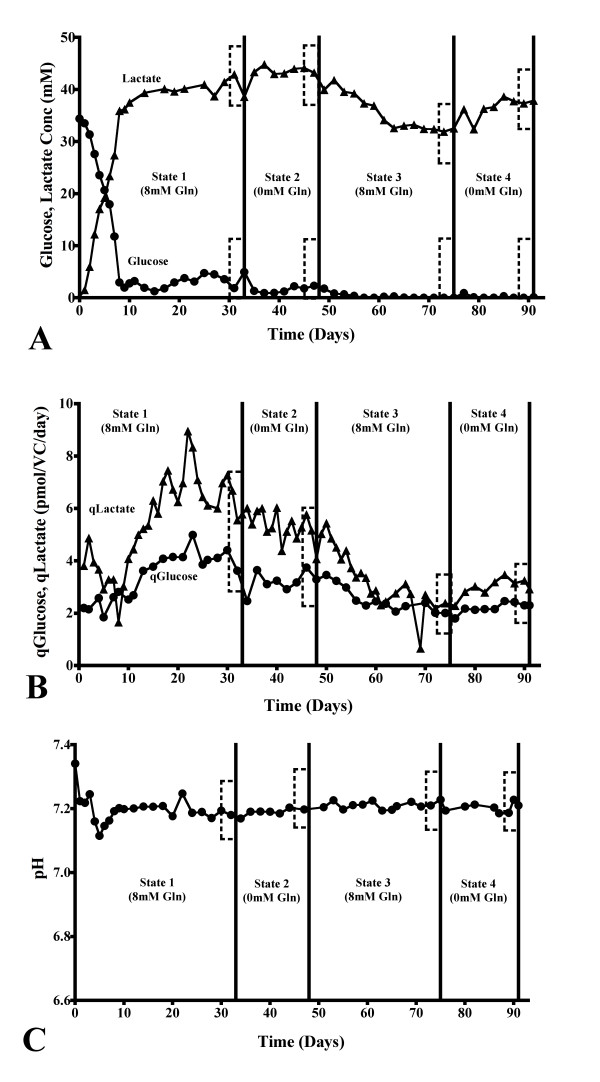
**The effects of glutamine on glucose, lactate and pH in bioreactor continuous culture**. The concentrations and rates of consumption and production of glucose and lactate during continuous culture with varying glutamine concentrations in the culture medium (A and B, respectively). The extracellular culture pH during continuous culture with varying glutamine concentrations in the culture medium (C). Boxes are used to illustrate the steady state examined. Panels A, B and C, n = 8.

With the flow of culture medium in the continuous culture set-up, the steady state concentration of glucose was approximately 2 mM at states 1 and 2 (Figure 3A). Lactate stabilized at a concentration of 40 mM at these same steady states (Figure 3A). The concentration of both glucose and lactate decreased significantly at steady states 3 and 4 to approximate values of 0 mM and 35 mM, respectively (p < 0.05). There was no difference in the rates of glucose consumption and lactate production at steady states 1 and 2 of the continuous cultivation, although these values decreased significantly at states 3 and 4 (p < 0.001, Figure 3B). However, the continuous cultivation was maintained at pH 7.2 by the addition of CO_2 _and NaOH (Figure 3C).

### Metabolism of glutamine, glutamate and ammonium

A minor difference in the intended and measured initial concentration of glutamine in all set-ups was observed, and can be attributed to error range of the Nova Bioprofile.

**Figure 4:**
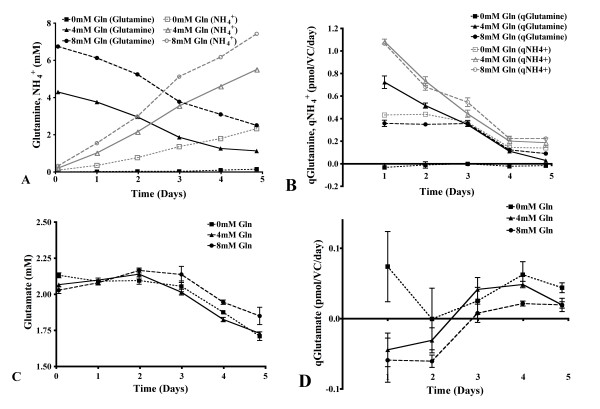
**Glutamine and glutamate metabolic profiles in batch shake flasks**. The extracellular concentration of glutamine with 0 mM, 4 mM and 8 mM glutamine supplementation and ammonium (identical symbols, colored grey) in batch shake flask cultivation (A). The rate of glutamine consumption and ammonium production with varied glutamine concentrations in batch shake flask cultivation (symbols identical to previous, B). The extracellular concentration and rate of consumption of glutamate during batch shake flask cultivation (identical symbols, C and D respectively). Panels A, B, C and D, n = 6.

The extracellular concentrations of glutamine and ammonium were significantly different at all time points for the shake flask cultivation, and significant concentrations of ammonium were produced in the absence of glutamine supplementation (p < 0.001, Figure 4A). There was no significant difference in the rate of glutamine consumption or ammonium production when glutamine was supplied to shake flask cultures at concentrations of 4 mM and 8 mM (Figure 4B). However, both these rates were significantly lower on days 1 through 3 of culture without glutamine (p < 0.01). All cultures had a similar concentration of glutamate at each time point, although the concentration was decreased significantly on days 4 and 5 of culture with 0 mM and 4 mM glutamine (p < 0.05, Figure 4C). Despite the obvious trend on day 1, the large range of values for glutamate consumption for the cultures without glutamine supplementation resulted in no significant difference in the rate of consumption at any time point for any culture (Figure 4D).

**Figure 5:**
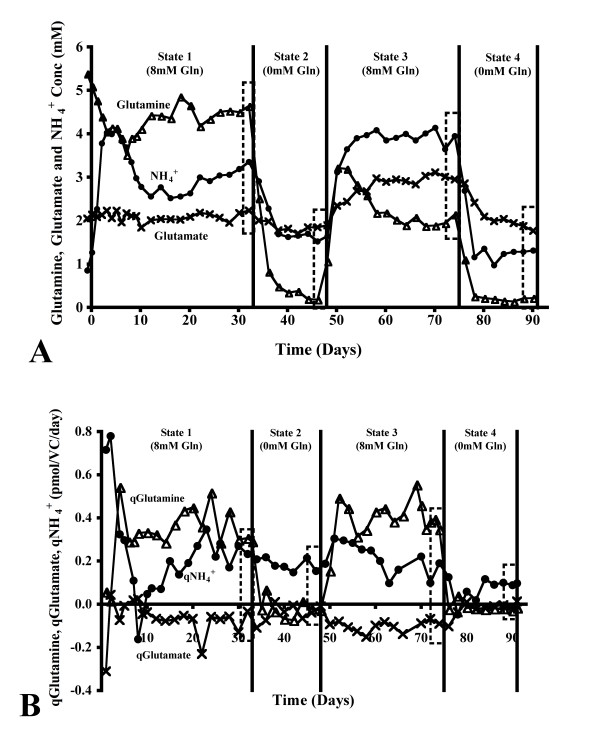
**Glutamine and glutamate metabolic profiles in bioreactor continuous culture**. The extracellular concentration and rate of consumption and production of glutamine, glutamate and ammonium during continuous culture with varying glutamine concentrations in the culture medium (A and B, respectively). Boxes are used in both instances to illustrate the steady state examined. Panels A and B, n = 8.

For the continuous cultivation, the extracellular concentration of glutamine decreased significantly between steady state 1 to 3 (p < 0.001), the concentration of glutamate increased at state 3 only (p < 0.001), while the concentration of ammonium was significantly different at each steady state (p < 0.05, Figure 5A). However, a similar rate of glutamine consumption was noted at states 1 and 3, no significant difference in the rate of glutamate production was noted during any part of the continuous cultivation, while the rate of ammonium production was seen to decrease during the cultivation time (p < 0.01 for the comparison between steady states 1 and 4, Figure 5B).

### Productivity of HCG

**Figure 6:**
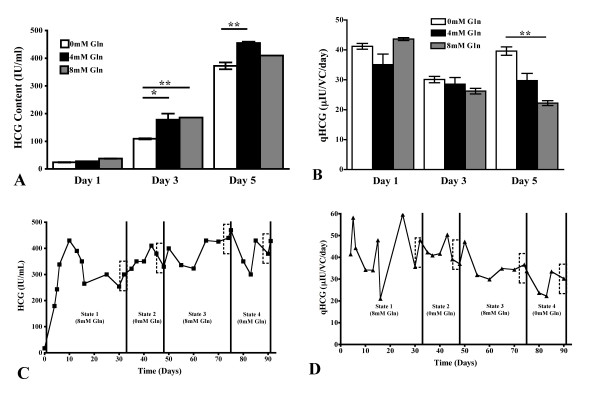
**Glutamine controls HCG production in shake flask and continuous culture systems**. The total content and specific productivity of HCG on days 1, 3 and 5 of shake flask culture with 0 mM, 4 mM and 8 mM glutamine supplementation (white, black and grey, respectively, A and B, * = p < 0.05, ** = p < 0.01). Total content and specific productivity of HCG during continuous cultivation with varying glutamine concentrations (C and D, respectively). Boxes are used to illustrate the steady state examined. Panels A and B, n = 6; panels C and D, n = 3.

The total amount of HCG increased throughout batch culture at all glutamine concentrations to reach a maximal value of approximately 400 IU/ml on day 5. The amount of HCG at 0 mM glutamine was significantly lower than both glutamine supplemented cultures on day 3, although the value was only statistically different to the culture at 4 mM glutamine on day 5 (Figure 6A). The specific productivity of HCG was found to differ significantly on day 5 of shake flasks culture only, where the cells cultivated at 0 mM glutamine had a statistically higher productivity than the culture grown at 8 mM glutamine only (Figure 6B).

The total amount of HCG stabilized at approximately 300 IU/ml at each steady state of the continuous culture, although this increased significantly to 443 IU/ml at steady state 3, which was deemed to be statistically higher than state 1 only (p < 0.01, Figure 6C). No significant difference was noted in the specific productivity of the continuous culture, although a decreasing trend was noted (Figure 6D).

### Metabolic flux analysis at each steady state of continuous culture

**Figure 7:**
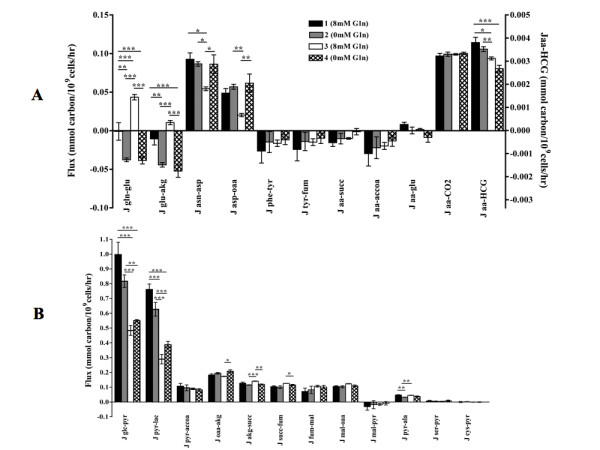
**Glutamine controls metabolic flux in bioreactor continuous cultures**. The changes in the main fluxes into the TCA cycle and through glycolysis and the TCA cycle at each steady state of continuous culture (A and B). Jaa-glu is the sum of the fluxes from histidine, arginine and proline to glutamate, Jaa-succ is the sum of the fluxes from methionine, isoleucine, threonine and valine to succinyl-CoA, Jaa-accoa is the sum of the fluxes from lysine, isoleucine, leucine and tyrosine to acetyl-CoA. Jaa-CO2 was assumed to have derived from amino acid metabolism only. The relative contribution of each amino acid towards the synthesis of HCG (or any recombinant protein) is described elsewhere (Gambhir et al., 2003). (* = p < 0.05, ** = p < 0.01, *** = p < 0.001; Panels A and B, n = 5).

At steady state 1, there was no net flux from glutamate to glutamine, although α-ketoglutarate is used for the formation of glutamate (Jgln-glu and Jglu-akg, Figure 7A). However, with the removal of glutamine from culture medium at state 2, there was a positive flux from α-ketoglutarate to glutamate to glutamine, indicating the production of glutamine at this state (Figure 7A). At state 3, this flux was reversed, with positive flux from glutamine to glutamate and α-ketoglutarate (Figure 7A). At steady state 4, where no glutamine is supplemented to the culture, the flux was the same as that observed at state 2, where α-ketoglutarate is converted to glutamate and glutamine (Figure 7A). A significant decrease in the flux from asparagine to aspartate to oxalaoacetate was observed at state 3 (Jasn-asp, Jasp-oaa, Figure 7A). There was a significant decrease in the flux from amino acids towards the synthesis of HCG from states 1 to 4 (Figure 7A).

With the transition from excess glucose concentrations of 2 mM at states 1 and 2 to limiting values of 0 mM at states 3 and 4, the flux from glucose to pyruvate and from pyruvate to lactate decreased significantly at these states (Jglc-pyr and Jpyr-lac, states 3 and 4, Figure 7B). However, there was no change in the flux from pyruvate to the TCA cycle at acetyl-CoA at any steady state (Jpyr-accoa, Figure 7B). There was a significant decrease in the flux from oxaloacetate to α-ketoglutarate at state 3, although the subsequent flux through the TCA cycle, from α-ketoglutarate to succinyl-CoA was increased (Joaa-akg and Jakg-succ, Figure 7B). The flux from pyruvate to alanine decreased significantly at state 2 (Figure 7B).

### Intracellular content of nucleotides and sugar nucleotides

**Figure 8:**
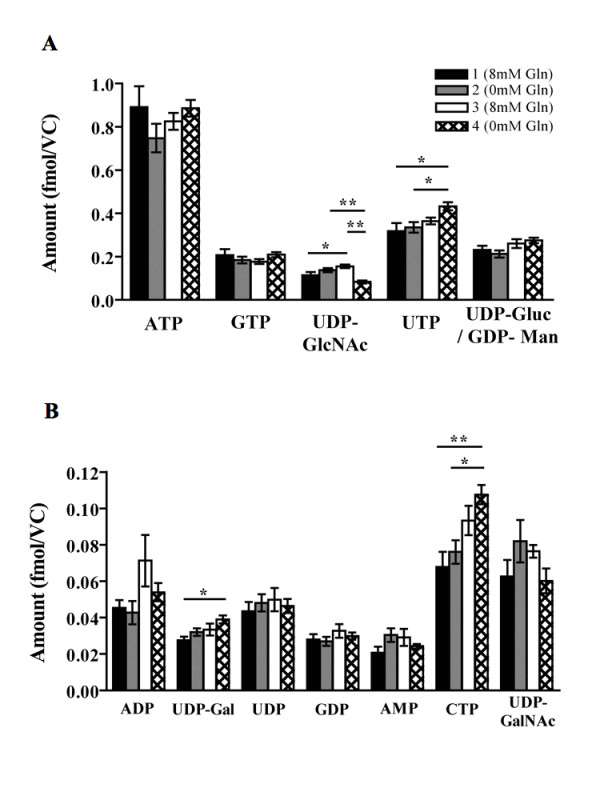
**Glutamine controls nucleotides and sugar nucleotide concentrations in bioreactor continuous culture**. The intracellular content of nucleotides and sugar nucleotides at each steady state of continuous culture (A and B, * = p < 0.05, ** = p < 0.01, *** = p < 0.001; Panels A and B, n = 6).

Significant differences in the intracellular content of nucleotides and sugar nucleotides were noted at state 4 of continuous culture. The concentration of UDP-GlcNAc was statistically reduced, while UTP, UDP-Gal and CTP were increased (Figure 8A and 8B).

### Glycosylation of HCG

**Figure 9:**
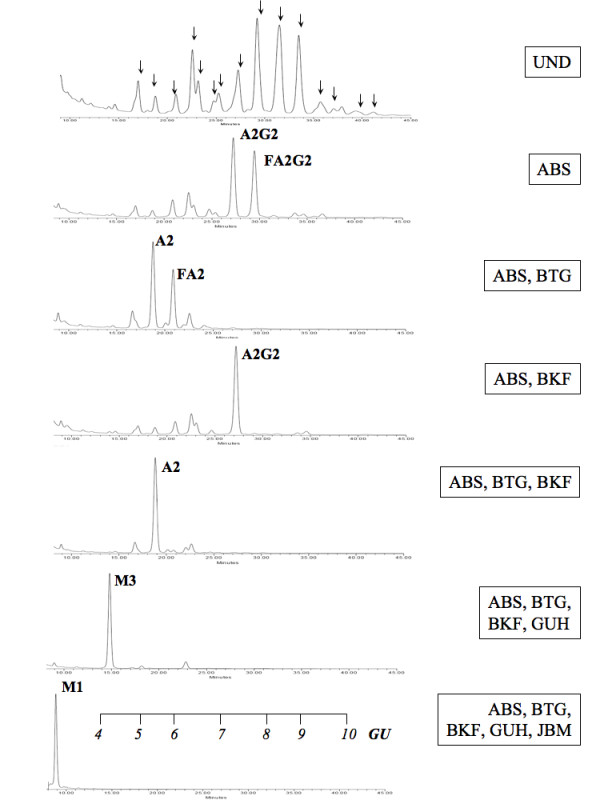
**HPLC analysis of the recHCG glycan pool**. The HCG glycan pool was subjected to exoglycosidase digestions in order to assign structures (A). UND represents the undigested pool of glycans, where the arrows denote the peaks referred to in Tables 1 and 2. This pool was digested with combinations of sialidase A (ABS), β-galactosidase (BTG), α-fucosidase (BKF), hexosaminidase (GUH) and mannosidase (JBM). The composition of each large peak is described alongside. The structural abbreviations for glycans are as follows; all complex structures were assumed to have 2 core GlcNAc and 3 mannose residues, F represents the number of core linked fucose residues, Man(*x*) the number (*x*) of mannose residues on core GlcNAcs, A(*x*), the number (*x*) of antennae (GlcNAc) on a trimannosyl core, G(*x*), the number (*x*) of galactose residues β-linked to the antennae, S(*x*) the number (*x*) of sialic acids on antennae. For example, NeuAc_2_Gal_2_GlcNAc_2_Man_3 _+ Fuc would be represented as FA2G2S2. A GU (glucose unit) scale is shown for clarity.

The *N*-linked glycan pool of HCG separated into 15 peaks, comprised of 23 structures with NP-HPLC (Figure 9, UND). (Structural abbreviations are described in the legend for Figure 9). Digestion with a series of exoglycosidase enzymes allowed the identification of the glycan structures within each peak. For example, the two major peaks observed in NP-HPLC after sialidase digestion were found to comprise of A2G2 and FA2G2 (ABS digestion, Figure 9). Inclusion of β-galactosidase with this pool of glycans resulted in a decrease in GU value of each of these peaks, corresponding to the removal of two galactose residues from each structure (A2 and FA2, ABS, BTG digestion). However, inclusion of α-fucosidase resulted in the formation of one peak of A2G2 (proportionally increased area, ABS, BKF digestion). Digestion with both fucosidase and β-galactosidase reduced this structure to A2 and a similar decrease in GU value (ABS, BTG, BKF digestion). Further digestion to the basic mannose structures was achieved with use of a hexosaminidase and mannosidase (ABS, BTG, BKF, GUH and ABS, BTG, BKF, JBM digestions).

A number of changes in the *N*-linked glycosylation of HCG were noted on days 1, 3 and 5 of shake flask culture. On day 1 of cultivation at 0 mM glutamine, the proportion of peaks 4 and 8 increased, while on day 3, peaks 2 and 4 were increased, which demonstrates that a higher proportion of neutral structures were produced under these conditions (p < 0.01, Table 1). By day 5, there were a number of changes that resulted in an overall pattern of decreased sialylation, fucosylation and antennarity and an increase in the proportion of neutral structures at 0 mM glutamine (p < 0.05, illustrated in peaks 10 through 15). These same changes in glycosylation were seen at steady state 4 of continuous culture, where glutamine was not supplemented to the growing cultures (p < 0.05, the mean percentage areas of A1G1 and M5 increased while peaks 10 through 15 decreased, Table 2).

**Table 1:** Glutamine controls recHCG glycoforms in shake flask cultures.

Peak **No**.	Glycan Structure	Day 1 0 mM Gln	Day 3 0 mM Gln	Day 5 0 mM Gln	Day 1 4 mM Gln	Day 3 4 mM Gln	Day 5 4 mM Gln	Day 1 8 mM Gln	Day 3 8 mM Gln	Day 5 8 mM Gln
**1**	A1	15.8 ± 7.4	18.8 ± 3.4	11.5 ± 0.8	7.8 ± 7.6	11.5 ± 3.2	10.0 ± 1.0	6.5 ± 5.8	12.3 ± 3.3	10.8 ± 0.1

**2**	A2	0.4 ± 0.0	**0.7 ± 0.0**	0.6 ± 0.1	0.2 ± 0.0	0.4 ± 0.0	0.9 ± 0.1	0.3 ± 0.0	0.5 ± 0.0	0.8 ± 0.0

**3**	A1G1	2.9 ± 0.4	3.7 ± 0.4	**4.2 ± 0.2**	0.9 ± 0.4	1.2 ± 0.4	1.6 ± 0.0	1.1 ± 0.2	1.5 ± 0.1	1.7 ± 0.1

**4**	M5	**8.0 ± 0.5**	**8.0 ± 0.5**	**8.3 ± 0.4**	1.8 ± 0.1	2.8 ± 0.0	3.8 ± 0.0	1.9 ± 0.0	3.3 ± 0.1	4.1 ± 0.1

**5**	A2G1, A1G1S1	2.5 ± 0.5	3.0 ± 0.0	3.2 ± 0.3	2.4 ± 0.3	2.6 ± 0.3	3.6 ± 0.3	2.3 ± 0.5	3.0 ± 0.1	3.8 ± 0.0

**6**	A2G1S1	2.0 ± 0.6	1.8 ± 0.3	2.8 ± 0.1	0.9 ± 0.1	1.5 ± 0.0	1.5 ± 0.1	1.0 ± 0.2	1.3 ± 0.1	1.4 ± 0.1

**7**	FA2G1	0.8 ± 0.5	1.0 ± 0.0	0.9 ± 0.2	1.2 ± 0.3	1.1 ± 0.1	2.4 ± 0.1	1.2 ± 0.2	1.2 ± 0.1	2.5 ± 0.0

**8**	A2G2, FA2G1S1	**13.5 ± 1.3**	12.6 ± 1.2	16.4 ± 0.1	7.3 ± 0.4	9.3 ± 0.0	8.3 ± 0.5	7.8 ± 0.2	8.5 ± 0.2	7.9 ± 0.4

**9**	FA2G2, A2G2S1	20.7 ± 2.0	19.6 ± 1.5	22.6 ± 0.7	22.5 ± 0.5	23.7 ± 0.1	21.3 ± 0.4	23.9 ± 0.1	22.6 ± 0.8	20.3 ± 0.6

**10**	A2G2S2, FA2G2S1	**19.5 ± 1.2**	**17.1 ± 1.1**	**17.6 ± 0.4**	32.6 ± 5.3	26.1 ± 0.9	26.3 ± 0.2	32.7 ± 4.6	27.1 ± 1.3	25.6 ± 0.4

**11**	A3G2S1, FA2G2S2	**8.8 ± 0.2**	**9.7 ± 0.7**	**7.6 ± 0.3**	14.6 ± 2.3	12.4 ± 1.6	13.2 ± 1.6	14.1 ± 0.3	12.3 ± 1.1	13.2 ± 0.9

**12**	A3G2S2, A3G3S1	**1.9 ± 0.5**	**1.5 ± 0.1**	**1.7 ± 0.1**	2.5 ± 0.2	2.4 ± 0.2	2.2 ± 0.3	2.4 ± 0.3	2.0 ± 0.1	2.5 ± 0.1

**13**	FA3G3S1, A3G3S2	**1.3 ± 0.2**	**1.0 ± 0.1**	**1.3 ± 0.1**	2.1 ± 0.1	2.1 ± 0.2	1.9 ± 0.1	1.9 ± 0.1	1.8 ± 0.0	2.3 ± 0.0

**14**	A3G3S3, FA3G3S2	**1.1 ± 0.0**	**0.9 ± 0.1**	**1.1 ± 0.0**	2.3 ± 0.4	1.9 ± 0.2	2.0 ± 0.1	2.2 ± 0.3	1.8 ± 0.1	2.0 ± 0.0

**15**	FA3G3S3	**0.6 ± 0.0**	**0.5 ± 0.2**	**0.4 ± 0.1**	0.8 ± 0.0	0.9 ± 0.2	1.0 ± 0.2	0.6 ± 0.2	0.8 ± 0.2	1.1 ± 0.0

The relative structures and proportions of each peak of the HCG *N*-linked glycan NP-HPLC pool and on day 1, 3 and 5 of batch culture with varying concentrations of glutamine. Numbers in bold indicates significant difference, p < 0.05, n = 6.

**Table 2:** Glutamine controls recHCG glycoforms in bioreactor continuous culture.

Peak **No**.	Glycan Structure	Steady State 1 (8 mM Gln)	Steady State 2 (0 mM Gln)	Steady State 3 (8 mM Gln)	Steady State 4 (0 mM Gln)
**1**	A1	2.16 ± 0.66	4.57 ± 2.64	4.58 ± 2.05	4.44 ± 1.34

**2**	A2	1.13 ± 0.27	1.30 ± 0.21	2.31 ± 0.50	1.36 ± 0.24

**3**	A1G1	1.32 ± 0.24	1.90 ± 0.40	2.64 ± 0.60	**5.89 ± 0.97**

**4**	M5	4.56 ± 0.44	4.72 ± 0.61	6.77 ± 1.01	**14.70 ± 3.37**

**5**	A2G1, A1G1S1	3.60 ± 0.19	3.45 ± 0.06	3.81 ± 0.35	4.66 ± 0.81

**6**	A2G1S1	1.42 ± 0.19	1.63 ± 0.24	1.65 ± 0.22	2.41 ± 0.22

**7**	FA2G1	3.53 ± 0.47	3.54 ± 0.23	3.78 ± 0.42	2.40 ± 0.27

**8**	A2G2, FA2G1S1	7.39 ± 0.59	8.38 ± 0.91	9.29 ± 1.31	**14.33 ± 2.08**

**9**	FA2G2, A2G2S1	18.31 ± 0.98	18.03 ± 0.84	16.56 ± 0.36	22.06 ± 2.92

**10**	A2G2S2, FA2G2S1	26.90 ± 0.73	24.51 ± 1.04	20.90 ± 2.18	**14.55 ± 1.15**

**11**	A3G2S1, FA2G2S2	20.99 ± 1.60	20.14 ± 1.42	16.02 ± 2.29	**8.87 ± 0.84**

**12**	A3G2S2, A3G3S1	2.95 ± 0.23	2.79 ± 0.26	5.54 ± 1.28	**2.23 ± 0.18**

**13**	FA3G3S1, A3G3S2	2.67 ± 0.25	2.40 ± 0.19	3.77 ± 0.62	**1.23 ± 0.17**

**14**	A3G3S3, FA3G3S2	1.97 ± 0.11	1.70 ± 0.15	1.50 ± 0.29	**0.57 ± 0.06**

**15**	FA3G3S3	1.07 ± 0.14	0.96 ± 0.13	0.90 ± 0.14	**0.30 ± 0.04**

The relative structures and proportions of each peak of the HCG *N*-linked glycan NP-HPLC pool with varying concentrations of glutamine at each steady state of continuous culture are shown. Numbers in bold indicates significant difference, where each steady state was compared side-by-side, p < 0.05 and n = 3.

## Discussion

### Batch culture

Differences in growth rate, glucose metabolism and culture pH have been shown to alter the structure of *N*-linked glycans [11-13]. Changes in the *N*-linked glycosylation of HCG were noted in our batch shake flasks cultured at 0 mM glutamine (Table 1). This overall pattern of decreased sialylation, fucosylation and antennarity of the *N*-linked glycans, and increase in the proportion of neutral structures was accompanied by a number of metabolic changes under these conditions. The rates of cell growth, glucose consumption and lactate production were all reduced in the early stages of culture at 0 mM glutamine, while these values increased above those determined for the glutamine supplemented cultures on the last day of culture (Figures 1, 2). The rate of ammonium production was also significantly lower at 0 mM glutamine on days 1 through 3 of culture, while the specific productivity of HCG was increased on day 5 (Figures 4, 6). The culture pH was higher under these conditions (Figure 2), consequently, the lack of glutamine resulted in a number of metabolic and glycosylation changes in this cell culture set-up. This provided a good basis for further investigation of the effect of glutamine on *N*-linked glycosylation in a more stable culture system, in this case with use of continuous culture.

### Continuous culture

A simple illustration of the increased stability of this culture system is seen when comparing the culture pH in batch and continuous culture (Figure 2C, 3C). With this bioreactor set-up, many process parameters are controlled (e.g. pH, pO_2_, pCO_2_), and the continuous culture method ensures that the metabolism of the cells stabilized after a defined period of time (e.g. qGluc, qLac, qGln, qGlu, qNH_4_^+^, Figure 3 and 5).

After an initial period of adaptation, the cells settled into four steady states. The changes in the *N*-linked glycosylation of HCG during shake flask cultivation at 0 mM glutamine were also present at steady state 4 of continuous culture (Figure 9C). The rates of glucose consumption, lactate production and the flux from glucose to pyruvate and lactate decreased at states 3 and 4 (Figures 3, 7). Additionally, the intracellular content of UDP-GlcNAc decreased at state 4 (Figure 8).

**Figure 10:**
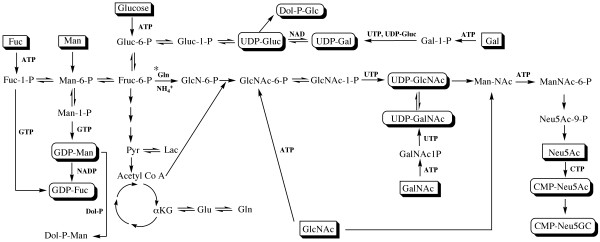
**Sugar nucleotide metabolism**. The formation of activated sugar nucleotides is intrinsically linked to cellular metabolism. For example, the conversion of glucose to pyruvate (Pyr) during glycolysis also forms fructose-6-phosphate (Fruc-6-P), which contributes to the accumulation of the UDP-GNAc pool (UDPGlcNAc and UDP-GalNAc), in a similar manner to glutamine (Gln) and ammonium (NH_4_^+^). The content of this pool has a direct effect on the proportion of sialic acid precursors (such as CMP-Neu5Ac) and may impact the antennarity of glycans since UDP-GlcNAc is a substrate for GlcNAc transferase enzymes, which catalyse the addition of glycan chains. Glycan donors and monosaccharides are shown in rectangles. Additional abbreviations are noted elsewhere.

The antennarity and sialylation of *N*-linked glycans is altered by varying the intracellular content of the UDP-GNAc sugars i.e. UDP-GlcNAc and UDP-GalNAc [14-17]. Under cell culture conditions, high glutamine and/or ammonium concentrations contribute to the accumulation of the pool of UDP-GNAc by the combined action of the enzymes glucosamine-6-phosphate isomerase (GPI) and glucosamine-6-phosphate synthetase (GPS) to form glucosamine-6-phosphate from fructose-6-phosphate (reaction marked with an asterisk, Figure 10). GlcNAc transferase enzymes catalyse the addition of new glycan chains to the basic biantennary N-linked glycan structure and require UDP-GlcNAc as a substrate. Altered concentrations of UDP-GlcNAc impact on the sialylation of glycoproteins since this sugar nucleotide is a necessary precursor for the synthesis of CMP-NeuAc, the activated sugar donor for the sialic acid termed Neu5Ac. Additionally, the accumulation of the UDP-GlcNAc pathway requires an adequate glycolytic flux, since fructose-6-phosphate is formed during glycolysis before conversion to UDP-GNAc [18].

Therefore, the reduction in sialylation and antennarity of HCG may be related to the decreased intracellular content of UDP-GlcNAc caused by a lack of glutamine coupled with low glycolytic flux. The reasons for the decrease in fucosylation were not examined, but it is possible that the reduction in glycolytic flux has an impact, since fructose-6-phosphate is interconverted to fucose-1-phosphate, a precursor for the formation of the GDP-fucose sugar donor. Glutamine limitation has previously been shown to induce an increase in nucleotide levels [3], observed in the case of UTP and CTP in this study. In addition, the proportion of each *N*-linked glycan found in shake flask and continuous cultivation was largely similar, though the amount of underprocessed glycans was reduced in shake flasks (peaks 2, 6 and 7, Tables 1 and 2).

The results of the metabolic flux analysis illustrate the mechanisms by which these cells adapt to the lack of glutamine, since this amino acid is necessary for the synthesis of nucleotides and nicotinamide co-enzymes, in addition to the previously described role in the formation of activated sugars. Under conditions of excess glucose, there was no net flux from glutamine to the TCA cycle, suggesting that glutamine was primarily used in these other pathways (Jgln-glu, steady state 1, Figure 7). At steady state 2 and 4, where glutamine is not supplied to culture medium, there is positive flux from α-ketoglutarate in the TCA cycle to glutamate and glutamine, indicating that glutamine was formed under these conditions to maintain the glutamine status of the cell (Jglu-akg, Jgln-glu, Figure 7).

**Figure 11:**
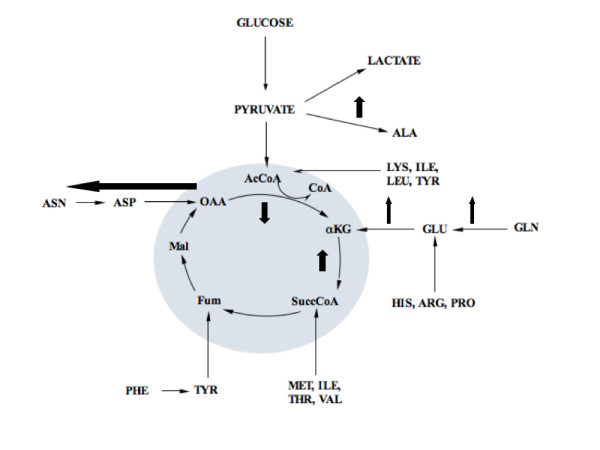
**Schematic of the changes in metabolic flux at steady state 3**. Small black arrows indicate normal flux direction, while emboldened black arrows indicate the change in metabolic flux at this steady state. In this case, there was an increase in the flux towards production of alanine from pyruvate and asparagine and aspartate from oxaloacetate (likely caused by the increased ammonium concentration, see text for details). This resulted in a decrease in the flux from oxaloaceate to α-ketoglutarate. However, the flux through the TCA cycle was maintained by an increase in the flux from glutamine to glutamate and α-ketoglutarate, which resulted in an increase in the mean net flux from α-ketoglutarate to succinyl-CoA in the TCA cycle. The remaining calculated fluxes in the TCA cycle were comparable to those seen at the remaining steady states. Abbreviations are noted elsewhere.

A distinct set of changes to the fluxes around the TCA cycle was seen at steady state 3 (Figure 7, schematic illustration Figure 11). Firstly, the flux through glycolysis was reduced at this state (Figure 7B). The formation of amino acids, such as alanine and aspartate, have been suggested to occur as a result of either "overflow metabolism" during inefficient cellular metabolism [19], or as a method to attempt to detoxify ammonium [20]. Transamination of glutamate maintains the toxic amino group within another amino acid, which prevents the formation of an additional ammonium ion. With the increased concentration of ammonium at this state (Figure 5A), it is possible that the increased flux from pyruvate to alanine and from oxaloacetate to asparagine and aspartate was a result of metabolic diversion to reduce the ammonium content (Jpyr-ala, Jasn-asp and Jasp-oaa, Figure 7). The net result of the increase in flux away from the TCA cycle at oxaloacetate was a reduction in the subsequent flux to α-ketoglutarate (Joaa-akg, Figure 7B). If this reduction in flux through the TCA cycle was maintained past this point, it is likely that cellular metabolism may be impaired, since this pathway is a key node for the provision of a range of metabolic intermediates. However, in this state, a positive flux from glutamine to glutamate and α-ketoglutarate occurred, which increased the mean net flux from α-ketoglutarate to succinyl-CoA in the TCA cycle, when compared to the previous state (Jgln-glu, Jglu-akg, Jakg-succ, Figure 7).

This indicates that the aim to replicate data by the sequential cultivation method was unsuccessful, however, these results suggest a case of steady state multiplicity - a difference in cell metabolism despite the use of identical dilution rate and medium composition in a continuous culture [6,21,22]. In one case, sequentially reducing the dilution rate of a continuous culture of hybridoma cells resulted in more efficient metabolism of glucose, and some elements of this altered metabolism were maintained when the cells were subsequently returned to the initial high dilution rate [22]. In another series of investigations, it was shown that use of fed-batch culture conditions preceding the onset of medium flow for continuous culture of a different hybridoma cell line can alter the resulting steady state achieved [6,21]. In this case, the flux through glycolysis and the TCA cycle and in the metabolism of glutamine was altered between steady states 1 and 3. The reasons for this are uncertain, however, the reduction in the extracellular concentration of glucose and glycolytic flux may be the key factor. It is also possible that this was merely a cellular adaptation to the loss of glutamine. Further studies into the mechanisms by which these alternative metabolic states are reached are necessary, since recent work has shown that significant changes in the enzymes of central metabolism occur at both the genomic and proteomic level, in cases of induced steady state multiplicity in a continuous culture system [23].

### Glutamine and productivity of HCG

The productivity of HCG was significantly increased on day 5 of batch shake flask culture with 0 mM glutamine supplementation (Figure 6B). The growth rate of cells was significantly higher for this culture, when compared to glutamine-supplemented culture, although the total amount of HCG was not (Figures 1B, 6A). The maintenance of a higher growth rate for a longer time may explain why the productivity was similarly increased.

Despite the lack of significance in the trend of changing productivity during the continuous culture, there was a statistical decrease in the flux from amino acids towards the synthesis of HCG (Figures 6, 7). A reduction in the productivity of recombinant proteins is not unexpected following extended culturing of a cell line [24]. However, the productivity of tPA in a continuous culture of CHO cells has been shown to vary with the concentration of glucose in the culture medium [25,26]. Consequently, the reduction in the extracellular concentration of glucose at states 3 and 4 may have resulted in this decrease in productivity.

## Conclusions

This work has illustrated that reducing glutamine concentrations to 0 mM in a batch shake flask resulted in reduced sialylation, fucosylation and antennarity, and, increased proportions of neutral *N*-linked glycans attached to HCG. By its nature, batch cultivation resulted in variations in metabolism during the culture time, such as growth rate, the rate of consumption and production of key metabolites and recombinant protein, and, also in some important culture conditions such as pH. With use of a continuous culture set up, growth rate and culture pH were controlled while the glutamine concentration in culture medium was altered between 8 mM and 0 mM. Additionally, a metabolic steady state was attained; the consumption and production rates of key metabolites stabilized after a key length of time. By determining the intracellular content of nucleotides and sugar nucleotides, the mechanisms for these changes were explored. It was demonstrated that lack of glutamine coupled with reduced glycolytic flux caused a reduction in the sialylation and antennarity of the *N*-linked glycans attached to HCG, an event mediated by the decrease in the intracellular pool of UDP-GlcNAc. The use of metabolic flux analysis at each steady state illustrated the metabolic diversions that occurred in the presence of varying glutamine concentrations, and highlighted a case of steady state multiplicity. The occurrence of multiple steady states under the same culture conditions suggests the potential to guide cells into key metabolic states that may improve the characteristics of recombinant proteins. Consequently, this method of coupling use of continuous culture with analytical techniques to probe the mechanisms of changing *N*-linked glycosylation is effective, and lends itself towards a range of other applications.

## Abbreviations

Aa: Amino acid; 2AB: 2-Aminobenzamide; AcCoA: Acetyl coenzyme; akg: α-Ketoglutarate; CHO: Chinese hamster ovary cells; CMP-NeuAc: CMP-N-acetylneuraminic acid; Fuc: Fucose; Fum: Fumarate; Gal: Galactose; GDP: Guanosine diphosphate; GDP-Man: GDP-mannose; Glc: Glucose; Gln: Glutamine; Glu: Glutamate; HCG: Human chorionic gonadotrophin; Lac: Lactate; Mal: Malate; Neu5Ac: N-acetylneuraminic acid; OAA: Oxaloacetate; Pyr: Pyruvate qX: Rate of consumption or production of X; SuccCoA: Succinyl-Co-A; tPA: Tissue plasminogen activator; UDP-Gal: UDP-galactose; UDP-GalNAc: UDP-N-acetylgalactosamine; UDP-Glc: UDP-glucose; UDP-GlcNAc: UDP-N-acetylglucosamine; UDP-GNAc: UDP-GlcNAc/UDP-GalNAc.

## Competing interests

The authors declare that they have no competing interests.

## Authors' contributions

SCB carried out the studies and drafted the manuscript. TVDL CJMS, WMJVG and GPD conceived of the study, and participated in its design and coordination. GPD oversaw the project. NOD and PMR assisted in the interpretation of glycan analysis results. All authors read and approved the final manuscript.
